# Pre-Treatment with Ten-Minute Carbon Dioxide Inhalation Prevents Lipopolysaccharide-Induced Lung Injury in Mice via Down-Regulation of Toll-Like Receptor 4 Expression

**DOI:** 10.3390/ijms20246293

**Published:** 2019-12-13

**Authors:** Shih-En Tang, Shu-Yu Wu, Shi-Jye Chu, Yuan-Sheng Tzeng, Chung-Kan Peng, Chou-Chin Lan, Wann-Cherng Perng, Chin-Pyng Wu, Kun-Lun Huang

**Affiliations:** 1 Division of Pulmonary and Critical Care Medicine, Department of Internal Medicine, Tri-Service General Hospital, National Defense Medical Center, Taipei 11490, Taiwan; msetang@gmail.com (S.-E.T.); kanpeng@mail.ndmctsgh.edu.tw (C.-K.P.); wperng@ms27.hinet.net (W.-C.P.); 2 Institute of Aerospace and Undersea Medicine, National Defense Medical Center, Taipei 11490, Taiwan; shuyu0321@gmail.com; 3 Division of Rheumatology, Immunology and Allergy, Department of Internal Medicine, Tri-Service General Hospital, National Defense Medical Center, Taipei 11490, Taiwan; d1204812@mail.ndmctsgh.edu.tw; 4 Division of Plastic Surgery, Department of Surgery, Tri-Service General Hospital, National Defense Medical Center, Taipei 11490, Taiwan; m6246kimo@yahoo.com.tw; 5 Division of Pulmonary Medicine, Department of Internal Medicine, Taipei Tzuchi Hospital, New Taipei City 23142, Taiwan; bluescopy@yahoo.com.tw; 6 Department of Critical Care Medicine, Landseed Hospital, Taoyuan 32449, Taiwan

**Keywords:** acute respiratory distress syndrome, carbon dioxide, lipopolysaccharide, toll-like receptor 4

## Abstract

Various animal studies have shown beneficial effects of hypercapnia in lung injury. However, in patients with acute respiratory distress syndrome (ARDS), there is controversial information regarding the effect of hypercapnia on outcomes. The duration of carbon dioxide inhalation may be the key to the protective effect of hypercapnia. We investigated the effect of pre-treatment with inhaled carbon dioxide on lipopolysaccharide (LPS)-induced lung injury in mice. C57BL/6 mice were randomly divided into a control group or an LPS group. Each LPS group received intratracheal LPS (2 mg/kg); the LPS groups were exposed to hypercapnia (5% carbon dioxide) for 10 min or 60 min before LPS. Bronchoalveolar lavage fluid (BALF) and lung tissues were collected to evaluate the degree of lung injury. LPS significantly increased the ratio of lung weight to body weight; concentrations of BALF protein, tumor necrosis factor-α, and CXCL2; protein carbonyls; neutrophil infiltration; and lung injury score. LPS induced the degradation of the inhibitor of nuclear factor-κB-α (IκB-α) and nuclear translocation of NF-κB. LPS increased the surface protein expression of toll-like receptor 4 (TLR4). Pre-treatment with inhaled carbon dioxide for 10 min, but not for 60 min, inhibited LPS-induced pulmonary edema, inflammation, oxidative stress, lung injury, and TLR4 surface expression, and, accordingly, reduced NF-κB signaling. In summary, our data demonstrated that pre-treatment with 10-min carbon dioxide inhalation can ameliorate LPS-induced lung injury. The protective effect may be associated with down-regulation of the surface expression of TLR4 in the lungs.

## 1. Introduction

Acute respiratory distress syndrome (ARDS) develops most commonly in the context of severe sepsis, particularly when caused by infection with Gram-negative bacilli such as *Escherichia coli* [1,2,3]. The overall mortality of ARDS remains high, despite advances in the treatment of ARDS. The current mainstay of treatment for sepsis is limited to antibiotic therapy. Research focusing on the prevention of ARDS and identifying patients at risk of developing ARDS is necessary [4,5].

Hypercapnia and hypercapnic acidosis (HCA) exerts multiple important effects in lung injury and acute respiratory failure, which may be beneficial or deleterious to multiple biological pathways [6,7]. Previous ARDS studies have demonstrated that permissive hypercapnia is associated with lower hospital mortality [8,9]. The protective ventilation strategy through reducing tidal volume can improve survival of ARDS patients [10,11]. Moreover, laboratory studies have documented protective effects of HCA induced by adding inspired carbon dioxide in animal models of lung injury induced by free radicals [12], sepsis [13,14,15,16,17], ischemia-reperfusion [18,19,20,21], or excessive lung stretch [22,23,24].

However, HCA is not without risks. The safety of HCA in the setting of a live bacterial infection, such as pneumonia, remains a significant concern. Long-term exposure to HCA may impair the host response to an invading pathogen, permit bacterial proliferation, and ultimately worsen lung injury [25]. HCA stimulates endoplasmic reticulum stress in endothelial cells [26]. HCA also impairs lung epithelial wound healing after lung injury [27]. There is controversial information regarding the effect of hypercapnia on ARDS outcomes. In patients with ARDS, two recent studies in a large population of mechanical ventilation patients showed higher mortality associated with hypercapnia [28,29]. In ARDS patients, severe hypercapnia (PaCO_2_ > 50 mmHg) was associated with higher mortality and more organ failures [28]. Another retrospective analysis including over 250,000 ARDS patients receiving mechanical ventilation showed that patients who developed hypercapnic acidosis (pH < 7.35, PaCO_2_ > 65 mmHg) during the first 24 h of mechanical ventilation had significantly higher mortality [29]. Nonetheless, the timing and duration of HCA administration could be critical for shifting the balance in favor of the potential HCA benefits in lung injury. In this study, we investigated the effects of pre-treatment with short-term (10 or 60 min) of inhaled carbon dioxide on lipopolysaccharide (LPS)-induced lung injury in mice.

## 2. Results

### 2.1. Pre-Treatment with Inhaled Carbon Dioxide Suppressed LPS-Induced Weight Loss, Lung Injury, and Indicators of Oxidative Stress

**Figure 1:**
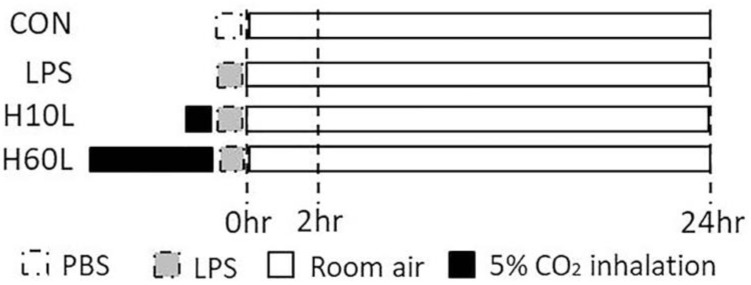
Schematic diagram of the first part of experimental protocol. The experiment was divided into two parts. The first part of the experiment included following 4 groups at 2 and 24 h (*n* = 6 in each group) after phosphate-buffered saline (PBS) or lipopolysaccharide (LPS) spray. Control (CON) group (Group 1): Intratracheal PBS (50 μL) was sprayed into the lungs of the mice in the CON group. LPS group (Group 2): Same concentration of LPS (2 mg/kg) was sprayed into the lungs of the mice in the various experimental groups. Two of the LPS groups also received carbon dioxide by exposure to 5% carbon dioxide for 10 min (Group 3: H10L group) or 60 min (Group 4: H60L group) before the LPS spray.

**Figure 2:**
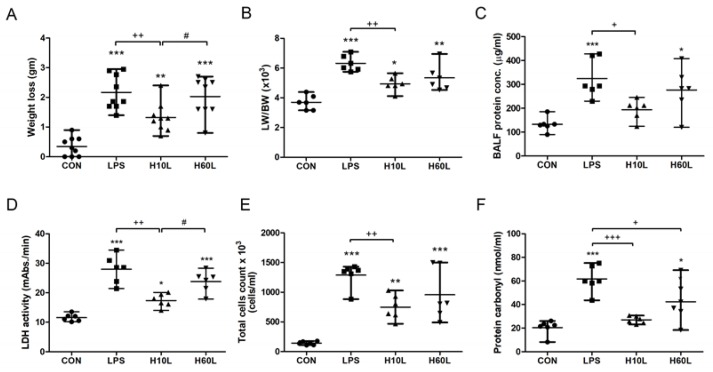
The effects of pre-treatment with inhaled carbon dioxide on lipopolysaccharide (LPS)-induced weight loss, pulmonary edema, inflammatory lung injury, inflammatory cell infiltration, and oxidative stress. C57BL/6 mice were challenged with LPS (2 mg/kg intratracheally) with or without pre-treatment with inhaled carbon dioxide for 10 min (H10L) or 60 min (H60L) before LPS administration. Control (CON) animals were treated with PBS solution. (**A**) Body weight loss was recorded 24 h after LPS administration (*n* = 9 per group). (**B**) The lung weight to body weight ratio (LW/BW) was recorded 24 h after treatment. Total protein concentration (**C**) and lactate dehydrogenase (LDH) activity (**D**) were measured in bronchoalveolar lavage fluid (BALF) collected 24 h after treatment. (**E**) Total cell count was determined in BALF. (**F**) The concentration of protein carbonyl in serum 24 h after LPS administration was measured by ELISA. The data are expressed as the mean ± standard deviation (SD) (*n* = 6 per group). * Significantly different from the CON group (* *p* < 0.05, ** *p* < 0.01, *** *p* < 0.001). ^+^ Significantly different from the LPS group (^+^ *p* < 0.05, ^++^ *p* < 0.01, ^+++^ *p* < 0.001). ^#^ Significantly different from the H10L group (^#^ *p* < 0.05).

To determine whether inhaled carbon dioxide had an anti-inflammatory effect in the lungs, mice were exposed to 5% carbon dioxide for 10 min or 60 min before LPS treatment (Figure 1). We evaluated the effect of inhaled carbon dioxide on the maintenance of body weight in LPS-induced lung injury in mice. Ten minutes, but not 60 min, of inhaled carbon dioxide attenuated the loss of body weight in LPS-induced lung injury in mice (Figure 2A). Intratracheal instillation of LPS caused lung injury, including pulmonary edema, microvascular protein leakage, and inflammatory cell infiltration. This lung injury was reflected in an increased ratio of lung weight to body weight (Figure 2B), in bronchoalveolar lavage fluid (BALF) protein concentration (Figure 2C), BALF lactate dehydrogenase (LDH) activity (Figure 2D), and BALF total cell count (Figure 2E). Carbon dioxide pre-treatment for 10 min, but not for 60 min, reduced the amount of pulmonary edema, microvascular protein leakage, cell damage, and inflammatory cell infiltration in the lungs considerably in the LPS-treated mice (Figure 2B–E). To examine whether pre-treatment with inhaled carbon dioxide could limit LPS-induced oxidative stress, the concentration of protein carbonyl in serum was measured by ELISA. Pre-treatment with inhaled carbon dioxide for 10 min or 60 min significantly reduced the concentration of protein carbonyl in serum from LPS-treated mice (Figure 2F).

### 2.2. Pre-Treatment with Inhaled Carbon Dioxide Reduced LPS-Induced Inflammatory Cytokine Levels

**Figure 3:**
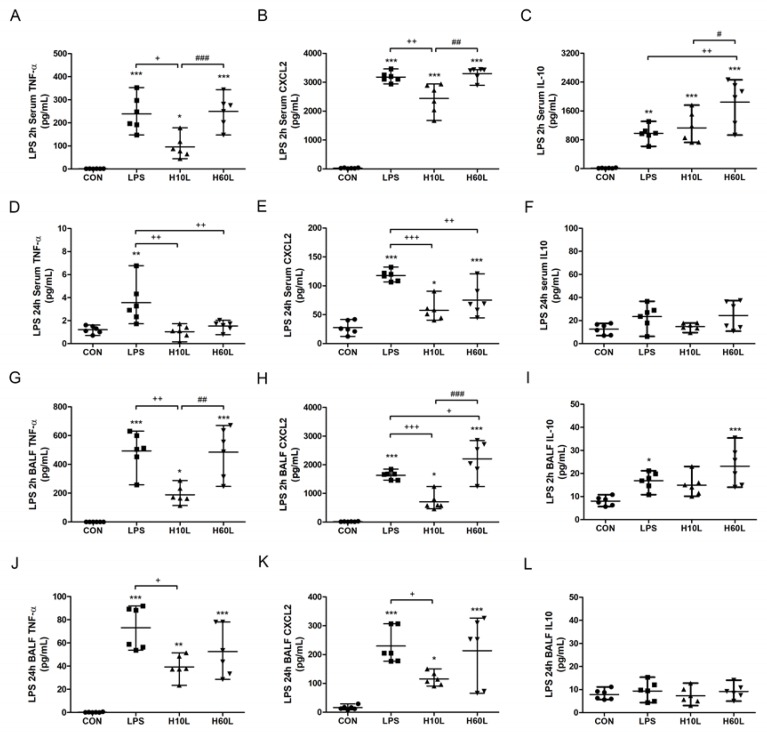
The effects of pre-treatment with inhaled carbon dioxide on lipopolysaccharide (LPS)-induced changes in TNF-α, CXCL2, and IL-10 concentrations. C57BL/6 mice were challenged with LPS (2 mg/kg intratracheally) with or without pre-treatment with inhaled carbon dioxide for 10 min (H10L) or 60 min (H60L) before LPS administration. Control (CON) animals were treated with PBS solution. The concentrations of TNF-α (**A**), CXCL2 (**B**), and IL-10 (**C**) in the serum at 2 h; TNF-α (**D**), CXCL2 (**E**), and IL-10 (**F**) in the serum at 24 h; TNF-α (**G**), CXCL2 (**H**), and IL-10 (**I**) in the bronchoalveolar lavage fluid (BALF) at 2 h; and TNF-α (**J**), CXCL2 (**K**), and IL-10 (**L**) in the BALF at 24 h after LPS administration were measured by enzyme-linked immunosorbent assay (ELISA). The data are expressed as the mean ± SD (*n* = 6 per group). * Significantly different from the CON group (* *p* < 0.05, ** *p* < 0.01, *** *p* < 0.001). ^+^ Significantly different from the LPS group (^+^ *p* < 0.05, ^++^ *p* < 0.01, ^+++^ *p* < 0.001). ^#^ Significantly different from the H10L group (^#^ *p* < 0.05, ^##^ *p* < 0.01, ^###^ *p* < 0.001).

LPS-induced production of TNF-α, a proinflammatory cytokine, chemokine CXCL2, and IL-10, an anti-inflammatory cytokine, in serum at 2 h (Figure 3A–C), in serum at 24 h (Figure 3D–F), in BALF at 2 h (Figure 3G–I), and in BALF at 24 h (Figure 3J–L). Both TNF-α and CXCL2, in the serum and BALF, at 2 and 24 h, were suppressed significantly by pre-treatment with inhaled carbon dioxide for 10 min, but not for 60 min. The shorter pre-treatment with inhaled carbon dioxide also substantially reduced the alveolar TNF-α (Figure 3J) and CXCL2 (Figure 3K) secretion at 24 h. Although TNF-α (Figure 3D) and CXCL2 (Figure 3E) in the serum at 24 h were suppressed by pre-treatment with inhaled carbon dioxide for 60 min, the concentration of CXCL2 (Figure 3H) in BALF at 2 h was significantly increased by pre-treatment with inhaled carbon dioxide for 60 min. Interesting, the concentration of IL-10, induced by LPS, in serum at 2 h was not suppressed by pre-treatment with inhaled carbon dioxide for 10 min, but was increased by pre-treatment with inhaled carbon dioxide for 60 min.

### 2.3. Pre-Treatment with Inhaled Carbon Dioxide Suppressed LPS-Induced Tissue Damage and Inflammatory Cell Infiltration

**Figure 4:**
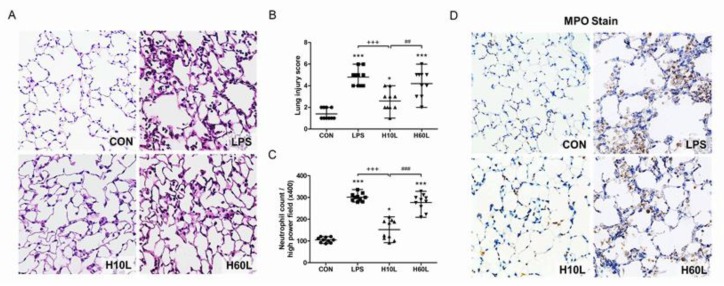
The effects of pre-treatment with inhaled carbon dioxide on lipopolysaccharide (LPS)-induced lung injury and inflammatory cell infiltration at 24 h. C57BL/6 mice were challenged with LPS (2 mg/kg intratracheally) with or without pre-treatment with inhaled carbon dioxide for 10 min (H10L) or 60 min (H60L) before LPS administration. Control (CON) animals were treated with PBS solution. (**A**) Representative images of hematoxylin and eosin-stained lung sections from one of the six mice per experimental group are presented. Original magnification = 40×. (**B**) The lung injury score and (**C**) neutrophil count were obtained from the images. The data are expressed as the mean ± SD. * *p* < 0.05, *** *p* < 0.001, compared with the CON group; ^+++^ *p* < 0.001, compared with the LPS group; ^##^ *p* < 0.01, ^###^ *p* < 0.001, compared with the H10L group. (**D**) Representative images of myeloperoxidase (MPO)-stained lung sections from one of the six mice per experimental group are presented. Original magnification = 40×.

**Figure 5:**
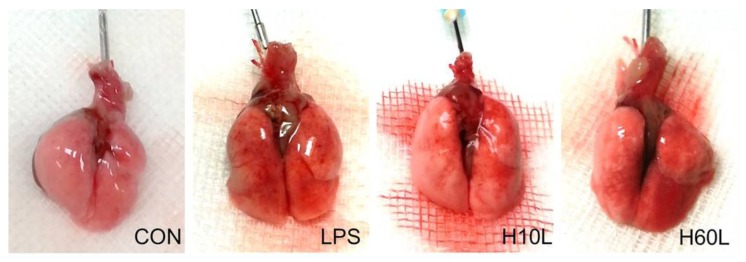
Gross lung pathology to demonstrate the effects of pre-treatment with inhaled carbon dioxide on lipopolysaccharide (LPS)-induced lung injury. C57BL/6 mice were challenged with LPS (2 mg/kg intratracheally) with or without pre-treatment with inhaled carbon dioxide for 10 min (H10L) or 60 min (H60L) before LPS administration. Control (CON) animals were treated with PBS solution. Representative images from one of the six mice per experimental group are presented.

Increased inflammatory responses were confirmed by microscopic examination, which showed septal thickening, alveolar edema, and increased inflammatory cell infiltration 24 h after LPS (Figure 4A). Ten minutes of pre-treatment with inhaled carbon dioxide reduced the lung injury score (Figure 4B) and LPS-induced neutrophil sequestration in the lungs considerably (Figure 4C). These results are consistent with the immunohistochemistry staining for myeloperoxidase (MPO) (Figure 4D). The overall appearance of congestion and edema 24 h after LPS was improved by pre-treatment with inhaled carbon dioxide for 10 min (Figure 5).

### 2.4. Inhaled Carbon Dioxide Pre-Treatment Inhibited LPS-Induced NF-κB Activation

**Figure 6:**
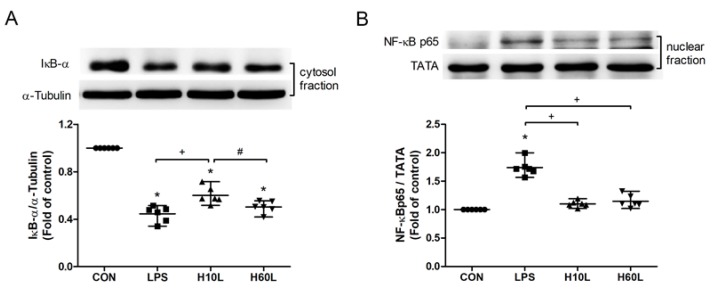
The effects of pre-treatment with inhaled carbon dioxide on lipopolysaccharide (LPS)-induced NF-κB activation. C57BL/6 mice were challenged with LPS (2 mg/kg intratracheally) with or without pre-treatment with inhaled carbon dioxide for 10 min (H10L) or 60 min (H60L) before LPS administration. Control (CON) animals were treated with PBS solution. The cytosolic levels of IκBα (**A**) and nuclear levels of NF-κB p65 (**B**) in lung tissues 2 h after LPS administration were analyzed by Western blot analysis and are expressed as fold change relative to α-tubulin and TATA, respectively. The data are expressed as the mean ± SD. * Significantly different from the CON group (*p* < 0.05). ^+^ Significantly different from the LPS group (*p* < 0.05). ^#^ Significantly different from the H10L group (*p* < 0.05).

The LPS-induced activation of the TLR4 and its downstream NF-κB signaling were investigated to determine the principles causing the anti-inflammatory actions of inhaled carbon dioxide. Pre-treatment with inhaled carbon dioxide for 10 min significantly inhibited LPS-induced IκBα degradation (Figure 6A) and NF-κB p65 nuclear translocation (Figure 6B) at 2 h.

### 2.5. Inhaled Carbon Dioxide Pre-Treatment and LPS Affected TLR4 Surface Expression

**Figure 7:**
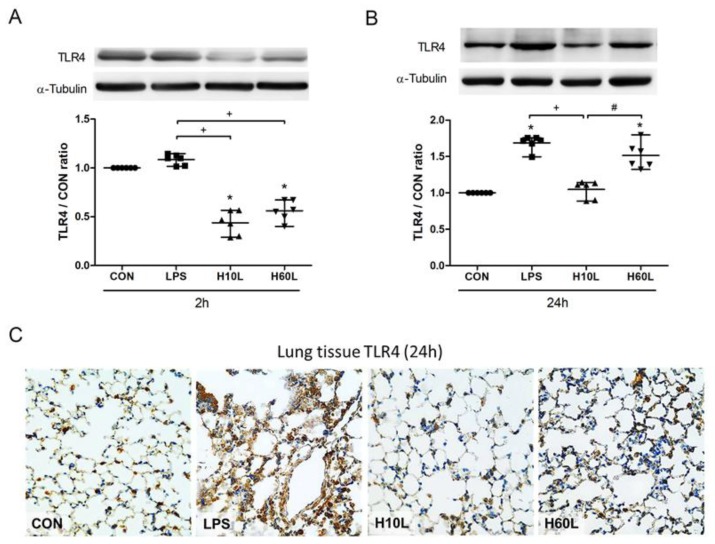
The effects of pre-treatment with inhaled carbon dioxide on lipopolysaccharide (LPS)-induced toll-like receptor 4 (TLR4) expression. C57BL/6 mice were challenged with LPS (2 mg/kg intratracheally) with or without pre-treatment with inhaled carbon dioxide for 10 min (H10L) or 60 min (H60L) before LPS administration. Control (CON) animals were treated with PBS solution. The surface expression of TLR4 was analyzed from the membrane fractions of lung tissues at 2 h (**A**) and 24 h (**B**) after LPS by Western blot analysis and is expressed as fold change relative to α-tubulin. The data are expressed as the mean ± SD. * Significantly different from the CON group (*p* < 0.05). ^+^ Significantly different from the LPS group (*p* < 0.05). ^#^ Significantly different from the H10L group (*p* < 0.05). (**C**) Representative images of TLR4-stained lung sections from one of the six mice per experimental group are presented. Original magnification = 40×.

To investigate the molecular mechanisms underlying the ability of inhaled carbon dioxide pre-treatment to attenuate LPS-induced lung injury, the expression of TLR4 protein in the lungs was assessed by Western blot analysis. The expression of TLR4 protein was maintained at 2 h (Figure 7A), but higher at 24 h (Figure 7B) after LPS administration. Pre-treatment with inhaled carbon dioxide for 10 min and for 60 min reduced TLR4 protein expression at 2 h after LPS stimulation. However, only pre-treatment with inhaled carbon dioxide for 10 min, but not for 60 min, significantly reduced LPS-induced TLR4 protein expression at 24 h after LPS stimulation (Figure 7B). The results were confirmed by immunohistochemical staining of TLR4 (Figure 7C). LPS increased but inhaled carbon dioxide pre-treatment suppressed TLR4 expression. The data suggest that the protective effects of inhaled carbon dioxide pre-treatment may be via suppression of TLR4 surface expression.

### 2.6. Inhaled Carbon Dioxide Affected TLR4 Surface Expression

**Figure 8:**
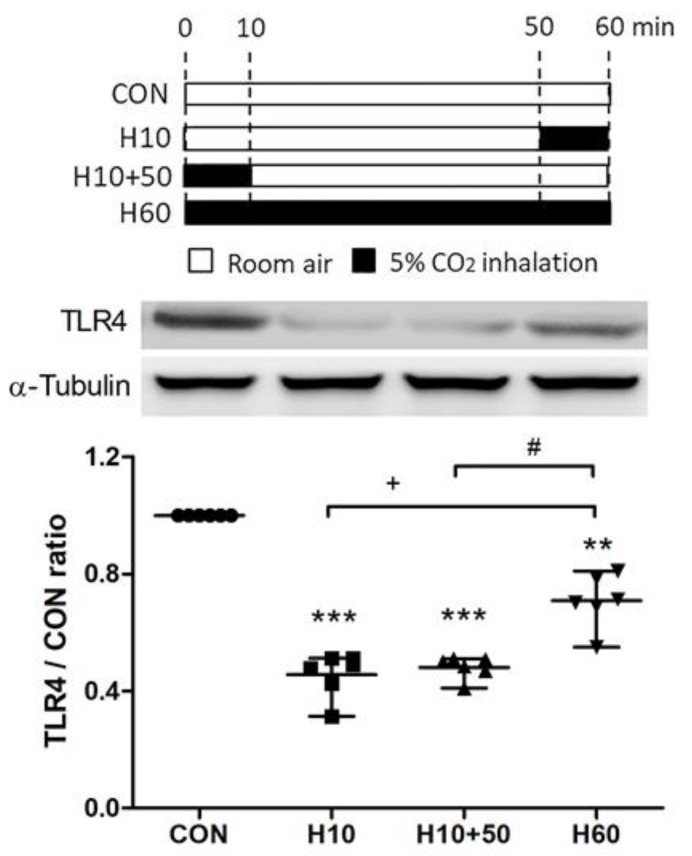
Schematic diagram of the second part of experimental protocol and the effects of carbon dioxide inhalation on toll-like receptor 4 (TLR4) expression. To investigate the effect of inhaled carbon dioxide on TLR4 expression in the lungs, the second part of experiment included following 4 groups at 1 h (*n* = 6 in each group). Control (CON) group (Group 1): C57BL/6 mice were treated with room air for 60 min. H10 group (Group 2): Mice were treated with room air for 50 min followed by inhaled carbon dioxide for 10 min. H10+50 group (Group 3): Mice were treated with inhaled carbon dioxide for 10 min followed by room air for 50 min. H60 group (Group 4): Mice were treated with inhaled carbon dioxide for 60 min. The surface expression of TLR4 was analyzed in the membrane fractions of lung tissues at the indicated time points by Western blot analysis and is expressed as fold change relative to α-tubulin. The data are expressed as the mean ± SD. * Significantly different from the CON group (** *p* < 0.01, *** *p* < 0.001). ^+^ Significantly different from the H10 group (*p* < 0.05). ^#^ Significantly different from the H10+50 group (*p* < 0.05).

A schematic diagram of the second part of the experimental protocol is shown in Figure 8. To investigate the effects of inhaled carbon dioxide on TLR4 surface expression, the expression of TLR4 protein in the lungs was assessed by Western blot analysis (Figure 8). The TLR4 protein surface expression was significantly reduced after exposure to inhaled carbon dioxide for 10 min (Group 2) and 60 min (Group 4). Interestingly, suppression of TLR4 surface expression by the 10-min inhaled carbon dioxide was sustained for at least 1 h (Group 3). However, the level of TLR4 protein surface expression after exposure to inhaled carbon dioxide for 60 min (Group 4) was higher than the other two groups that exposed to inhaled carbon dioxide for 10 min (Group 2) and inhaled carbon dioxide for 10 min with room air for 50 min (Group 3).

## 3. Discussion

We characterized the beneficial effects of inhaled carbon dioxide in a mouse model of LPS-induced lung injury. Instillation of LPS provoked leucocyte infiltration and cytokine production, NF-κB pathway activation, and increased alveolar capillary protein permeability, which suggests inflammatory lung injury. Pre-treatment with inhaled carbon dioxide for 10 min, but not 60 min, impeded NF-κB activation substantially, limited inflammatory cell invasion, and renewed the alveolar capillary integrity. These novel results suggest that pre-treatment with inhaled carbon dioxide for 10 min diminished LPS-induced lung damage by inhibiting the inflammatory reaction in the lungs.

These findings are consistent with those of other studies using a similar treatment protocol that revealed protective effects of inhaled carbon dioxide in animal models of LPS-induced lung injury [13] and pneumonia [14,15]. However, our study focused on prevention, and no additional carbon dioxide was given after the LPS administration. More studies focusing on preventing ARDS are needed to develop strategies to alter the clinical course of lung injury [4,5]. The pathophysiology of ARDS is complicated, and many theories have been proposed, such as the two-hit theory [30] and multiple-hit theory [31]. Our study suggests that inhaled carbon dioxide has potential as a strategy to block the progression of ARDS.

Previous studies have demonstrated the fundamental mechanisms by which HCA exerts beneficial effects, including attenuation of free radical production [12], nitric oxide-derived oxidant generation [13], apoptosis [18], NF-κB activation [20,24], and up-regulation of heme oxygenase-1 [21]. In our study, it was surprising to find that HCA induced by inhalation of carbon dioxide for only 10 min exerts anti-oxidative and anti-inflammatory effects. Moreover, inhalation of carbon dioxide for 10 min reflects a different mechanism for the down-regulation of TLR4 surface expression. A previous study showed the expressions of mRNA and protein of TLR4 in human bronchial epithelial cells was reduced after exposure for 24 h to hypercapnia (20% CO_2_) [32]. However, our study found that the protein expression of TLR4 was suppressed by inhalation of carbon dioxide for 10 min. This means that the TLR4 expressions of different durations of carbon dioxide inhalation may be controlled by different mechanisms. Further studies are needed to investigate the different mechanisms. TLR4 can sense pathogenic microorganisms by binding the bacterial product, LPS, to defend against invading pathogens [6]. However, TLR4 also can cause septic shock that involves overstimulation of the immune system [7]. Moreover, TLR4 is involved in various models of non-septic lung injury [8], such as oxidative stress [9], ischemia-reperfusion injury [10], and high stretch ventilation [11,12]. Thus, TLR4 signaling may serve as a therapeutic target for preventing or treating lung injury. Our study found that the down-regulation effect of TLR4 by 10 min of inhaled carbon dioxide could be prolonged to 60 min. This means the preventive time window may be extended to 1 h. Moreover, the protection offered by 10 min of inhaled carbon dioxide can last up to 24 h. The optimal duration of carbon dioxide inhalation between 10 min and 60 min need further investigation before clinical application.

Duration of exposure to HCA is critical for the application of inhaled carbon dioxide to prevent or limit lung injury. In bacterial pneumonia-related lung injury, the primary concern of HCA is impaired host innate immunity [33,34]. The protective effects of 6 h of therapeutic hypercapnic acidosis have been demonstrated in established severe bacterial pneumonia-induced lung injury [14,15]. However, another study showed that sustained hypercapnic acidosis lasting 48 h during pulmonary infection increased bacterial load and worsened lung injury [25]. O’Croinin et al. found no obvious protective effects of HCA on moderate-severity bacterial pneumonia-induced lung injury [35]. We found that the preventive effects of inhaled carbon dioxide were lost when the duration of inhalation extended to 60 min. This finding is consistent with a laboratory study showing that long-term carbon dioxide inhalation induced pulmonary inflammation [36]. Thus, prolonged exposure to HCA or inhalation of carbon dioxide may be harmful in many situations. This is consistent with a recent study of hospitalized patients with community-acquired pneumonia, which showed that both hypocapnia and hypercapnia have an increased need for intensive care unit admission and higher 30-day mortality [37]. In patients with chronic obstructive pulmonary disease, hypercapnia is associated with endothelial dysfunction [38] and poor prognosis [39,40].

Because of a possible dual role for TLRs in airway diseases, caution is needed when designing pulmonary TLR-based therapies [41]. A clinical study found that the extent of down-regulated TLR4 expression in the lungs was associated with the severity of emphysema and airflow limitation in smokers [42]. Modulation of TLR4 expression in the respiratory epithelium could cause an ineffective host response. This could lead to failure of the immune system to eradicate potentially pathogenic organisms, leaving the host susceptible to colonization and chronic inflammation. Additional investigation is needed to study the clinical implications of down-regulated TLR4 expression in patients with severe emphysema [42]. Whether or not down-regulated TLR4 expression is associated with hypercapnia requires confirmation [32].

Short-term inhalation of carbon dioxide has been applied in many clinical situations. Previous studies have shown that inhaled carbon dioxide is efficacious in eliminating Cheyne–Stokes respiration with central sleep apnea [43,44,45,46]. A clinical study found that hypercapnic hyperventilation, induced by insertion of additional airway dead space to keep the end-tidal carbon dioxide pressure close to 55 mmHg during hyperventilation, shortens the emergence time from isoflurane anesthesia [47]. Another study of children with febrile seizures showed that 5% carbon dioxide plus 95% oxygen was effective and safe for suppressing febrile seizures in children [48]. Thus, short-term exposed to a low concentration of inhaled carbon dioxide is a safe strategy for clinical application.

## 4. Materials and Methods

### 4.1. Animals

C57BL/6 adult male mice (8–10 weeks of age) were purchased from Charles River Technology in Taipei, Taiwan. All study animals were cared for in accordance with National Institutes of Health Guidelines (National Academy Press, Washington, DC, 1996).

The Institutional Animal Care and Use Committee at National Defense Medical Center and the National Science Council in Taipei, Taiwan approved the study (IACUC-14-101, 1 May 2014). The mice were subjected to carbon dioxide at ambient laboratory temperature in a large chamber fitted with automated controllers for carbon dioxide and oxygen. All mice were anesthetized during the procedures with intraperitoneal zolazepam–tiletamine (25 mg/kg of body weight; Zoletil^®^, Virbac, Carros, France) and xylazine (10 mg/kg of body weight; Rompun^®^, Bayer, Leverkusen, Germany).

*E. coli*-derived lipopolysaccharide (LPS) (*E. coli* serotype 0111:B4, Sigma Chemical Company, St. Louis, MO, USA) was prepared at a concentration of 1 mg/mL in phosphate-buffered saline (PBS). A MicroSprayer aerosolizer (IA-1C; Penn-Century Inc., Philadelphia, PA, USA) inserted into the trachea delivered the LPS solutions into the lungs of the mice.

### 4.2. Experimental Protocols

The mice were randomly assigned to the experimental groups. The experiment was divided into two parts. The first part of experiment included following 4 groups at 2 h (*n* = 6 in each group) and 24 h (*n* = 6 in each group) after PBS or LPS spray. Control (CON) group (Group 1): Intratracheal PBS (50 μL) was sprayed into the lungs of the mice in the control group. LPS group (Group 2): Same concentration of LPS (2 mg/kg) was sprayed into the lungs of the mice in the various experimental groups. Two of the LPS groups also received carbon dioxide by exposure to 5% carbon dioxide for 10 min (Group 3: H10L group) or 60 min (Group 4: H60L group) before the LPS spray. The primary outcome was the severity of lung damage. The secondary outcomes were the levels of inflammatory cytokines and oxidative stress markers, the loss of body weight, and the infiltrations of inflammatory cells. To investigate the effect of inhaled carbon dioxide without LPS on TLR4 expression in the lungs, the second part of experiment included following 4 groups (*n* = 6 in each group). CON group (Group 1) received room air for 60 min. H10 group (Group 2) received room air for 50 min followed by exposure to 5% carbon dioxide for 10 min. H10+50 group (Group 3) received carbon dioxide by exposure to 5% carbon dioxide for 10 min followed by room air for 50 min. H60 group (Group 4) received carbon dioxide by exposure to 5% carbon dioxide for 60 min.

### 4.3. Bronchoalveolar Lavage Fluid (BALF) Analysis

At the end of the experiment, BALF was collected. The chest was surgically opened, and the trachea was exposed. An intravenous infusion needle was inserted. After ligating the hilum of the right lung, the left lung was lavaged by irrigating it with two separate 0.5-mL aliquots of PBS. One BALF aliquot was used immediately to measure the total cell count, and another BALF aliquot was used for measurement of lactate dehydrogenase (LDH) activity after centrifugation at 200× *g* for 10 min. LDH activity in BALF was assessed as described previously [20]. The remaining fluid was centrifuged at 1000× *g* for 10 min, and the concentration of protein in the supernatant was assessed using a bicinchoninic acid protein assay kit (Thermo Fisher Scientific/Pierce, Rockford, IL, USA). The cell-free supernatant was stored at −80 °C for analysis of cytokines. The total leucocyte number was measured using an automated mammalian cell counter (ADAM-MC, Digital Bio, Seoul, Korea).

### 4.4. Protein Carbonyl Levels, Cytokine, and Chemokine Levels

Differences in serum protein carbonyl content were determined using a commercial kit (Carbonyl Protein Assay Kit, Cayman Chemical Company, Ann Arbor, MI, USA). Tumor necrosis factor-α (TNF-α), chemokine (C-X-C motif) ligand 2 (CXCL2), and Interleukin-10 (IL-10) in the serum or BALF were assessed using commercially available enzyme-linked immunosorbent assay (ELISA) kits (R&D Systems Inc., Minneapolis, MN, USA).

### 4.5. Histopathological Analysis

The mouse lung tissue was fixed in 10% neutral-buffered formalin for 24 h and then embedded in paraffin. The lung tissue samples were then sliced into 4-μm sections and stained with hematoxylin and eosin. Then, the severity of lung damage was scored as described previously [21]. The number of polymorphonuclear neutrophils in the lung interstitium was determined as the average number of polymorphonuclear neutrophils per high power field (×400). Ten fields were randomly examined by an observer unaware of the protocol. Within each field, lung injury was scored according to (1) infiltration or aggregation of neutrophils in the airspace or vessel wall, and (2) thickness of the alveolar wall. Each assessment was graded 0, 1, 2, or 3, for no, mild, moderate, or severe injury, respectively. The resulting two scores were added and presented as the lung injury score.

### 4.6. Immunohistochemistry Staining for Myeloperoxidase (MPO) and Toll-Like Receptor 4 (TLR4)

The paraffin-embedded mouse lung samples underwent immunohistochemical staining as described previously with minor modifications [21]. A polyclonal antibody against MPO (1:800 dilution; Thermo Fisher Scientific) and another polyclonal antibody against TLR4 (1:200 dilution; Bioss Inc., Woburn, MA, USA) were used.

### 4.7. Immunoblotting

Immunoblotting was performed as described previously [20]. Briefly, nuclear, cytoplasmic, and membrane proteins were separated from frozen lung tissue using the Nuclear/Cytosol/Membrane Subcellular Protein Fractionation Kit (Thermo Fisher Scientific). The fractionated proteins were transferred to blotting membranes and immunoblotted with antibodies against TLR4 (1:500 dilution; Bioss), inhibitor of NF-κB (IκBα), NF-κB p65, α-tubulin (1:1000 dilution; Cell Signaling Technology, Danvers, MA, USA), and TATA (for nuclear proteins, diluted 1:2,000; Abcam, Cambridge, MA, USA).

### 4.8. Statistical Analysis

All data are expressed as means ± standard deviations (SDs). Differences were evaluated using Student’s *t*-test, one-way analysis of variance, or two-way analysis of variance followed by Bonferroni’s post-hoc test where appropriate. Differences were considered significant at a *p*-value <0.05. All calculations were made using GraphPad Prism 6 software (GraphPad Software, San Diego, CA, USA).

## 5. Conclusions

In conclusion, we demonstrated that pre-treatment with inhaled carbon dioxide for 10 min, but not for 60 min, reduced LPS-induced lung inflammation considerably in mice. This protective action may be associated with down-regulation of TLR4 surface expression. The prophylactic application and optimal treatment timing for carbon dioxide inhalation require additional investigation.
